# Efficacy and safety of namilumab, a human monoclonal antibody against granulocyte-macrophage colony-stimulating factor (GM-CSF) ligand in patients with rheumatoid arthritis (RA) with either an inadequate response to background methotrexate therapy or an inadequate response or intolerance to an anti-TNF (tumour necrosis factor) biologic therapy: a randomized, controlled trial

**DOI:** 10.1186/s13075-019-1879-x

**Published:** 2019-04-18

**Authors:** Peter C. Taylor, Didier Saurigny, Jiri Vencovsky, Tsutomu Takeuchi, Tadashi Nakamura, Galina Matsievskaia, Barbara Hunt, Thomas Wagner, Bernard Souberbielle, for the NEXUS Study Group

**Affiliations:** 1 Botnar Research Centre, Nuffield Department of Orthopaedics, Rheumatology and Musculoskeletal Sciences, University of Oxford, Windmill Road, Oxford, OX3 7LD UK; 2 Takeda Development Centre, London, UK; 3 Institute of Rheumatology, Prague, Czech Republic; 4 Division of Rheumatology, Keio University School of Medicine, Tokyo, Japan; 5 Kumamoto Shinto General Hospital, Kumamoto, Japan; 6 Clinical Rheumatology Hospital #25, Saint-Petersburg, Russian Federation; 7 Statistics, Takeda International, Deerfield, IL USA; 8 Modeling and Simulation, Takeda Pharmaceuticals International GmbH, Zurich, Switzerland; 9 Present Address: GSK Medicines Research Centre, Stevenage, UK; 10 Present Address: Sangamo Therapeutics, London, UK; 11 Present Address: thinkQ2 AG, Baar, Switzerland

**Keywords:** Rheumatoid arthritis, Namilumab, GM-CSF

## Abstract

**Background:**

Namilumab (AMG203), an immunoglobulin G1 monoclonal antibody that binds with high affinity to granulocyte-macrophage colony-stimulating factor (GM-CSF), was evaluated in a phase II randomized, double-blind, placebo-controlled study to investigate the efficacy and safety in patients with rheumatoid arthritis (RA) with an inadequate response to methotrexate (MTX-IR) or anti-tumour necrosis factor therapy (TNF-IR).

**Methods:**

Subcutaneous namilumab (20, 80, or 150 mg) or placebo was administered at baseline and weeks 2, 6, and 10 in patients on stable background methotrexate therapy who were with MTX-IR or TNF-IR. Primary endpoint was mean change from baseline in the 28-joint Disease Activity Score, C-reactive protein version (DAS28-CRP) at week 12 comparing each of the three doses of namilumab to placebo. Safety and tolerability were assessed by adverse events (AEs) and pulmonary parameters. Results were analysed using the per-protocol population.

**Results:**

One hundred eight patients from Europe and Japan (48.4 ± 12.02 years old; 77.8% female; mean DAS28-CRP 5.60–5.79; rheumatoid factor/anti-citrullinated protein antibodies + 75%) were randomized to placebo or namilumab 20, 80, or 150 mg (*n* = 27, 28, 25, and 28, respectively). Ninety-two were MTX-IR; 16 were TNF-IR. At week 12, a statistically significant difference in DAS28-CRP (*p* = 0.005) was seen for namilumab 150 mg versus placebo and separation was seen as early as week 2 for namilumab 150 mg (*p* < 0.05), with higher ACR50 and response rates versus placebo at week 12. A dose-response effect was observed across the DAS28-CRP endpoint with separation versus placebo evident from week 2. The most common treatment-emergent AEs were nasopharyngitis (18.5%, 17.9%, 4.0%, 14.3%), dyspnoea (0.0%, 3.6%, 8.0%, 10.7%), bronchitis (7.4%, 3.6%, 4.0%, 3.6%), and headache (3.7%, 3.6%, 12.0%, 0.0%) for placebo and 20, 80, or 150 mg of namilumab, respectively. No serious infections were observed. One serious AE (myocardial infarction) was observed with 150 mg of namilumab. There was no apparent dose relationship for AEs. A biomarker-based disease activity score showed a dose-dependent decrease at week 12.

**Conclusions:**

This phase II study demonstrates the benefit of inhibiting macrophage activity targeting the GM-CSF for RA. The study met its primary endpoint with a clear dose-response effect. An acceptable tolerability profile was demonstrated over the 12-week study.

**Trial registration:**

ClinicalTrials.gov, NEXUS; NCT02379091, submitted November 28, 2014

**Electronic supplementary material:**

The online version of this article (10.1186/s13075-019-1879-x) contains supplementary material, which is available to authorized users.

## Introduction

Despite many treatments introduced for rheumatoid arthritis (RA), significant proportions of patients do not have their disease adequately controlled and thus are unable to achieve treatment goals [1–3]. There is a continuing need for the exploration and development of therapeutic strategies with novel mechanisms of action.

Granulocyte-macrophage colony-stimulating factor (GM-CSF) is a haematopoietic growth factor produced by a number of different cell types, including T cells, macrophages, mast cells, endothelial cells, smooth muscle cells, epithelial cells, and fibroblasts [4–9]. In patients with RA, GM-CSF is aberrantly overproduced [10–13]; GM-CSF levels are moderately elevated in the plasma and highly elevated in the synovial fluid [13, 14], particularly in the pannus at sites of cartilage erosion [15]. The contribution of GM-CSF to the development of RA has also been documented in various in vitro and in vivo mouse models [16–22]. Clinical proof of concept for GM-CSF–targeted therapy has been demonstrated in patients with RA for antibodies targeting the GM-CSF receptor (mavrilimumab) [23–26] and targeting soluble GM-CSF (MOR103) [27].

Namilumab (AMG203) is a human immunoglobulin G1 monoclonal antibody that binds with high affinity to the GM-CSF ligand, potently neutralizing GM-CSF [28]. Preclinical data showed that a surrogate mouse antibody of namilumab (22E9) neutralized GM-CSF, suppressed inflammation, and protected cartilage in an arthritis mouse model [29]. In a first-in-humans study, healthy volunteers showed that single doses of namilumab (up to 8.0 mg/kg) were generally well tolerated (Takeda; data on file). Subsequently, a phase Ib (PRIORA; clinicaltrials.gov ID No. NCT01317797), first-in-patient, multicentre, randomized, double-blind, placebo-controlled, dose-escalation study showed that subcutaneous namilumab was generally well tolerated and demonstrated preliminary evidence of efficacy, although patient numbers were small [30].

Here, we report the formal clinical proof-of-concept, dose-finding phase II global clinical trial in Europe and Asia of namilumab in patients with RA with either an inadequate response to background methotrexate (MTX-IR) or an inadequate response to an anti-tumour necrosis factor (TNF) biologic therapy (TNF-IR).

## Methods

### Patients

This study included patients with a diagnosis of adult-onset RA, defined by the 2010 American College of Rheumatology (ACR)/European League Against Rheumatism (EULAR) classification, who were being treated with stable doses of methotrexate of between 15 and 25 mg/week (between 6 and 16 mg/week in Japan) for at least 12 weeks prior to baseline (day 1), along with folic acid, and who had at least moderate disease activity (Disease Activity Score 28 [DAS28] ≥ 3.2), at least four swollen joints, and a visual analogue scale pain score > 40 mm. Concomitant nonsteroidal anti-inflammatory drugs with appropriate gastroprotection, low-dose corticosteroids (≤ 10-mg prednisone equivalence per day), and hydroxychloroquine (≤ 400 mg/day) or chloroquine (≤ 250 mg/day) were permitted at stable doses for at least 4 weeks before the first dose of the study drug. The MTX-IR population had been inadequately controlled by prior therapy comprising MTX alone or MTX in combination with other nonbiologic, disease-modifying antirheumatic drugs. The TNF-IR population had been inadequately controlled despite prior therapy with 1 TNF inhibitor. Subjects in this population had shown an inadequate response (insufficient initial response or loss of response after at least 12 weeks of treatment, and/or intolerance to a TNF inhibitor and/or due to safety, with the exception of the occurrence of serious adverse events).

Exclusion criteria included a history of or any current symptomatic or uncontrolled lung disease, active infection, or risk of infection or inflammatory joint disease other than RA or other systemic autoimmune disorders.

### Study design

Patients were randomized using an Interactive Web Response System in a 1:1:1:1 ratio and received either 20-, 80-, or 150-mg subcutaneous doses of namilumab or placebo at baseline, week 2, week 6, and week 10. The study was stopped at 12 weeks. Doses and administration frequency were based on phase Ia and phase Ib data and pharmacokinetic-pharmacodynamic modelling [30].

Institutional review boards or ethics committees at the participating investigational centres approved the study, which was conducted according to the principles set out in the Declaration of Helsinki, International Conference on Harmonisation Guidelines for Good Clinical Practice, and additional local regulations. The study was registered on clinicaltrials.gov as NEXUS (NCT02379091; registered March 4, 2015).

### Efficacy assessments

Efficacy assessments were performed at screening, baseline, week 2, week 6, week 10, and week 12. The primary endpoint was the mean change from baseline in DAS28-CRP (28-joint Disease Activity Score, C-reactive protein [CRP] version) at week 12 comparing each of the three dose levels of namilumab to placebo. This endpoint was analysed and controlled (balanced) for strata combined (TNF-IR and MTX-IR). Secondary endpoints included the proportion of subjects with an ACR20/50/70 response (≥ 20% improvement, ≥ 50% improvement, or ≥ 70% improvement in ACR score) at week 12 and change from baseline in DAS28-CRP at weeks 2 and 6. Additional endpoints included the proportion of patients achieving remission according to DAS28 (DAS28-CRP ≤ 2.6), improvements to the Health Assessment Questionnaire Disability Index (HAQ-DI) score, and change from baseline in quality of life as assessed by SF-36 v2 scales [31].

### Safety and tolerability assessments

Respiratory monitoring (chest radiograph, forced expiratory volume [FEV_1_], forced vital capacity [FVC], and MRC [Medical Research Council] breathlessness scale) was performed at screening and baseline and throughout the study because of the theoretical risk of developing pulmonary alveolar proteinosis when using therapeutic antibodies that target GM-CSF [32]. The protocol mandated referral to a pulmonologist for > 12% deterioration in FEV_1_ or FVC or for deterioration by > 5% decrease in oxygen saturation (SpO_2_) or an increase in MRC breathlessness scale score of 2 from baseline. These changes had to be reported as adverse events (AEs). Serum surfactant D (SP-D) and KL-6 (Krebs von den Lungen-6) levels, both established biomarkers for lung damage, were also measured. Other safety assessments included the incidence of AEs and serious AEs and routine laboratory testing.

### Pharmacodynamics

Pharmacodynamic assessments were performed of peripheral blood cytokines and other markers of inflammation, including disease activity (such as the multibiomarker disease activity [MBDA] Vectra DAscore [Crescendo Biosciences, South San Francisco, CA, USA] [33]) and structural damage biomarkers (such as C1M, a marker of tissue damage associated with structural disease progression [34] [Nordic Biosciences; Herlev, Denmark]).

### Statistical analyses

The following analysis sets were used for analysis and presentation of the study data: (1) all-patients-randomized set, consisting of all randomized subjects; (2) all-patients-treated set, consisting of all subjects in the all-patients-randomized set who took at least 1 dose of double-blind investigational medicinal product (IMP); (3) full analysis set (FAS), consisting of all subjects in the all-patients-treated set who had at least 1 valid post-baseline assessment of DAS28-CRP in the double-blind period up to week 12; and (4) per-protocol set (PPS), consisting of all subjects in the FAS who received a complete set of doses of IMP who completed their week 12 assessments and who did not have any major protocol violations that could have affected the efficacy assessment, such as patient populations who did not fulfil the protocol in terms of eligibility, interventions, and outcome assessment with a potential effect on efficacy (e.g. those who had used prohibited concomitant medications or those who were not on a stable background dose of methotrexate or corticosteroids).

Sample size calculations were based on the primary endpoint with the study powered for change in DAS28-CRP at 12 weeks. The total sample size of the study was expected to be approximately 100 (~ 25 subjects per arm). The study was designed as a proof-of-concept study to support dose finding but was not formally powered for dose-finding testing.

The treatment effect was implicitly assumed to be a 0.8 difference between placebo and test drug for improvement in DAS28-CRP for all active doses. According to Mandema et al. [35], a plot of the response against the dose reaches an upper plateau even at small doses for many biologic agents within RA, and an assumption of similar treatment effects for different doses therefore seems reasonable. The SD was assumed to be 1.15 [36]. The primary analysis was conducted using a detailed mixed-effect model repeated-measurement model. The test was the least squares (LS) means of the contrast between each of the active treatments and placebo.

For illustrative purposes, with these assumptions (difference from placebo = 0.80; SD of change from baseline = 1.15), 25 subjects per arm would provide 70% power to detect the treatment difference of 0.80 between active treatment group and placebo using a type I error level of 0.05 (two-sided, e.g. with no multiplicity adjustment).

## Results

### Patients

From a total of 171 patients screened, 108 were randomized between February 2015 and February 2016 in the study at 28 investigational sites in 7 countries (Bulgaria, Czech Republic, Poland, Russia, Spain, UK, and Japan). Of these, 88 were included in the PPS; 97 completed the 12-week period.

Patient demographics and baseline clinical characteristics are shown in Tables 1 and 2, respectively. The number of patients randomized per cohort was 27, 28, 25, and 28 for placebo and namilumab 20, 80, and 150 mg, respectively (2 patients were subsequently excluded from the FAS because of data integrity, which left 26, 28, 24, and 28 patients, respectively). For the PPS, the number of patients randomized per cohort was 20, 24, 23, and 21 for placebo and namilumab 20, 80, and 150 mg, respectively. The treatment groups were generally balanced in terms of baseline and disease characteristics (Table 2). There was a slightly higher proportion of male subjects taking 80 mg of namilumab and a lower proportion taking placebo compared with namilumab 20 and 150 mg, and there was a higher proportion of subjects taking corticosteroids in the 20-mg group compared with the other arms. Finally, there was a higher HAQ-DI score with placebo compared with the active treatment arm, and there was a higher mean CRP with placebo and with 150 mg of namilumab than with the other two study arms.

**Table 1:** Patient demographic characteristics

	Placebo (*N* = 27)	Namilumab		
		20 mg (*N* = 28)	80 mg (*N* = 25)	150 mg (*N* = 28)
Age, years	47.2 ± 13.45	46.1 ± 10.07	49.0 ± 9.60	51.3 ± 14.13
Female	23 (85.2)	22 (78.6)	17 (68.0)	22 (78.6)
Race				
Asian	6 (22.2)	4 (14.3)	3 (12.0)	5 (17.9)
White	21 (77.8)	24 (85.7)	22 (88.0)	22 (78.6)
Multiracial	0	0	0	1 (3.6)
BMI, kg/m^2^	23.75 ± 5.542	24.91 ± 5.210	27.16 ± 5.605	25.92 ± 6.313
BMI categories				
< 30 kg/m^2^	24 (88.9)	23 (82.1)	16 (64.0)	22 (78.6)
≥ 30 kg/m^2^	3 (11.1)	5 (17.9)	9 (36.0)	6 (21.4)
Smoking classification				
Nonsmoker	16 (59.3)	19 (67.9)	18 (72.0)	18 (64.3)
Ex-smoker	6 (22.2)	5 (17.9)	5 (20.0)	6 (21.4)
Current smoker	5 (18.5)	4 (14.3)	2 (8.0)	4 (14.3)
Region				
Ex-Japan	23 (85.2)	24 (85.7)	22 (88.0)	23 (82.1)
Japan	4 (14.8)	4 (14.3)	3 (12.0)	5 (17.9)

Values are mean ± SD or *n* (%). *BMI* body mass index

**Table 2:** Patient baseline clinical characteristics

	Placebo (*N* = 27)	Namilumab		
		20 mg (*N* = 28)	80 mg (*N* = 25)	150 mg (*N* = 28)
Mean duration of RA at screening, years	10.04 ± 8.880	9.02 ± 7.476	8.62 ± 8.501	7.35 ± 4.976
Positive rheumatoid factor at baseline	16 (59.3)	16 (57.1)	12 (48.0)	18 (64.3)
Anti-citrullinated peptide antibodies at baseline	20 (74.1)	19 (67.9)	12 (48)	21 (75)
Mean MTX dose at baseline, mg/week	15.73 ± 4.04	16.54 ± 4.741	16.06 ± 4.370	15.96 ± 4.238
Type of failure of prior treatment				
MTX-IR	23 (85.2)	23 (82.1)	22 (88.0)	24 (85.7)
TNF-IR	4 (14.8)	5 (17.9)	3 (12.0)	4 (14.3)
Corticosteroid use at baseline: yes	6 (22.2)	14 (50.0)	7 (28.0)	9 (32.1)
Mean DAS28-CRP score	5.71 ± 1.021	5.62 ± 0.931	5.63 ± 0.774	5.71 ± 1.122
Mean DAS28-ESR score	7.19 ± 1.033	7.07 (0.876)	6.99 (0.741)	7.2 (1.023)
Mean 66 swollen joint count	12.4 ± 8.78	12.8 (9.13)	15.8 (10.92)	13.6 (7.00)
Mean 68 tender joint count	23.0 ± 13.12	24.1 (11.91)	25.4 (12.49)	24.2 (13.24)
Mean patient’s global assessment of disease activity	68.6 ± 17.6	68.8 ± 15.17	65.9 ± 15.69	66.8 ± 16.27
Mean patient assessment of pain (VAS), mm	77.3 ± 17.43	75.0 ± 17.73	72.6 ± 13.7	69.3 ± 19.69
Mean physician’s global assessment of disease activity (mm)	68.6 ± 17.22	68.8 ± 15.17	65.9 ± 15.69	66.8 ± 16.27
Mean HAQ-DI	1.84 ± 0.67	1.61 ± 0.509	1.52 ± 0.516	1.49 ± 0.605
Median CRP, mg/L	7.91	3.95	6.29	9.41
Mean CRP, mg/L	17.12 ± 22.63	12.24 ± 15.4	8.92 ± 8.9	24.55 ± 60.5
Mean MBDA score	48.15 ± 17.515	47.92 ± 20.17	41.91 ± 15.47	48.77 ± 17.75
Mean C1M, ng/mL	35.45 ± 19.355	35.65 ± 24.99	35.36 ± 26.26	46.68 ± 45.56
SF-36 mental health	49.1± 17.92	50.4 ± 17.19	57.2 ± 18.08	50.6 ±18.24
SF-36 vitality	30.1 ± 17.51	31.5 ± 14.6	36.1 ± 14.35	31.7 ± 16.67

Values are mean ± SD or *n* (%) unless otherwise indicated. *CRP* C-reactive protein, *DAS28* Disease Activity Score 28, *ESR* erythrocyte sedimentation rate, *HAQ-DI* Health Assessment Questionnaire Disability Index, *MBDA* multibiomarker disease activity, *MTX* methotrexate therapy, *MTX-IR* inadequate response to methotrexate therapy, *RA* rheumatoid arthritis, *TNF-IR* inadequate response or intolerance to an anti-tumour necrosis factor biologic therapy, *VAS* visual analogue scale, *SF-36* 36-Item Short-Form Health Survey

**Fig. 1:**
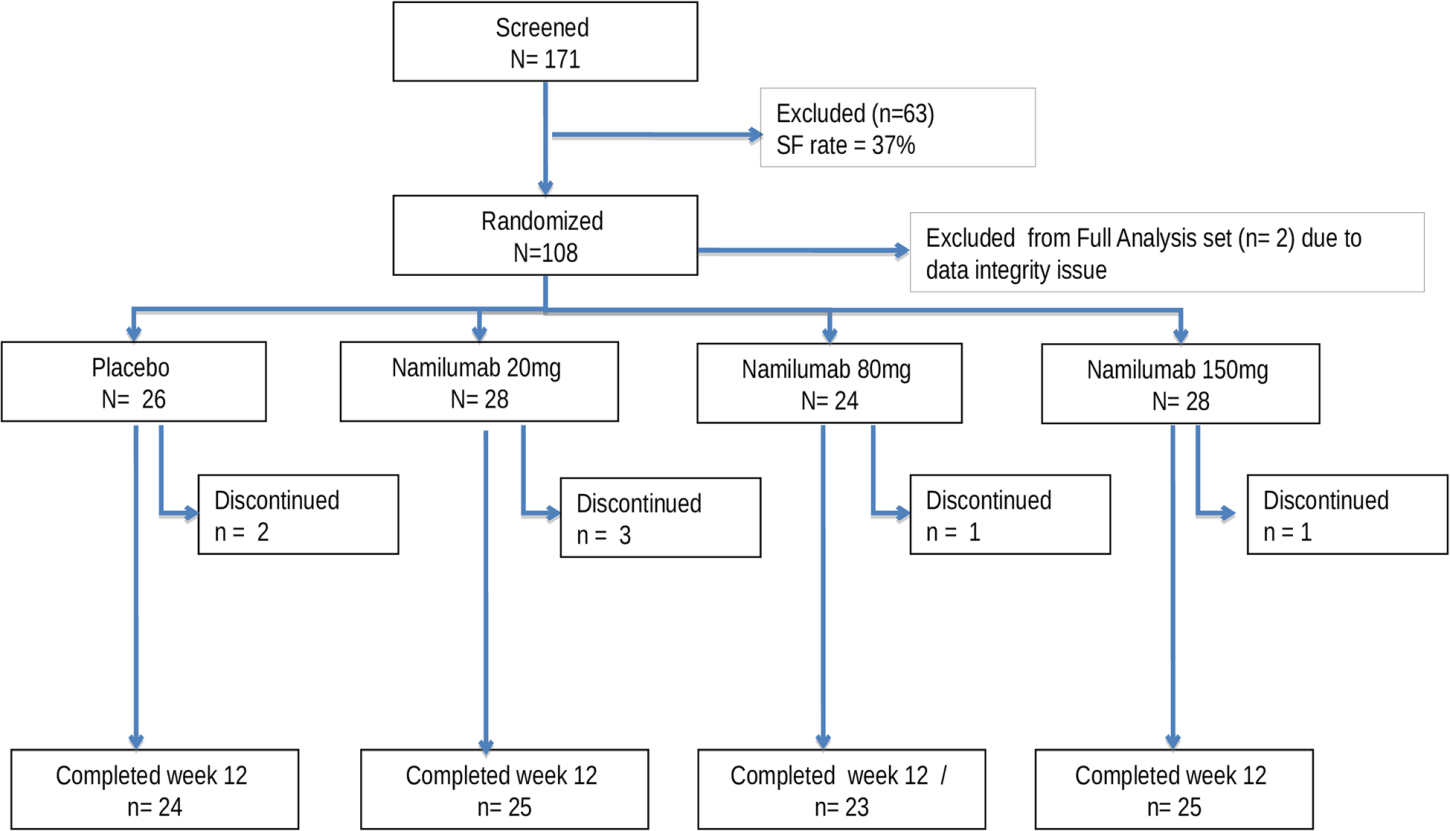
Subject disposition. The primary analysis was based on 106 subjects (full analysis set population) and 88 subjects (per-protocol set population). SF screening failure

Most of the patients completed the week 12 treatment, with only 7 withdrawing early (2 receiving placebo and 3, 2, and 1 receiving namilumab 20, 80, and 150 mg, respectively). Three of these early withdrawals were because of AEs (Fig. 1).

### Efficacy

**Fig. 2:**
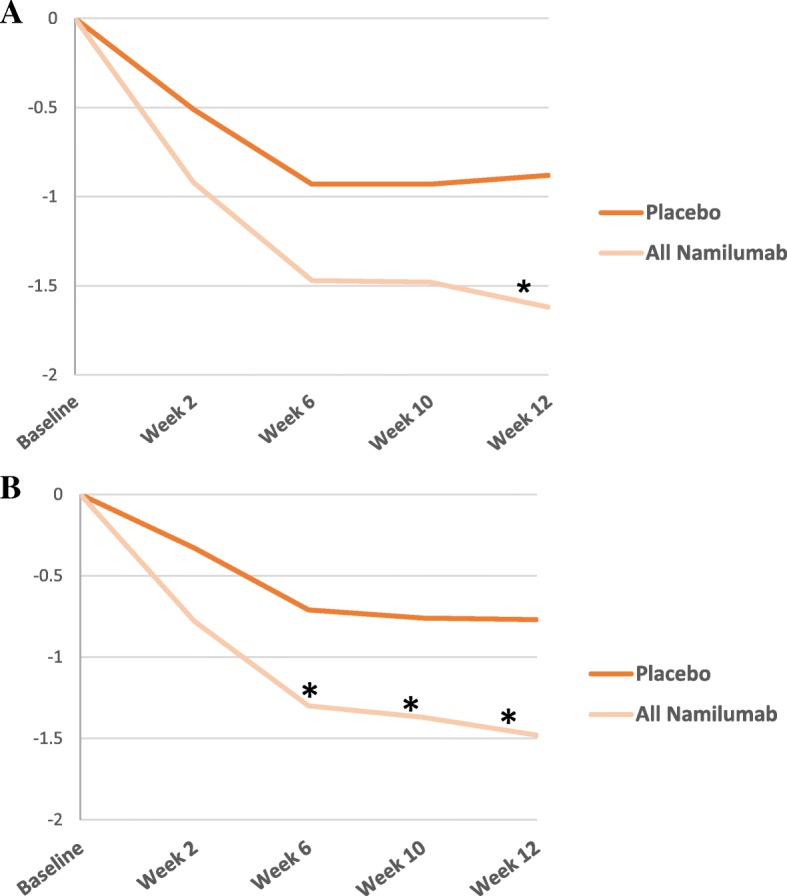
Analysis of change from baseline in DAS28-CRP score. Change from baseline in DAS28-CRP score by visit for the **a** per-protocol set [*p* < 0.05 are shown by asterisk; week 12, *p* = 0.022] and **b** full analysis set [*p* < 0.05 are shown by asterisk; week 6, *p* = 0.026; week 10, *p* = 0.031; week 12, *p* = 0.015]. DAS28-CRP 28-joint Disease Activity Score, C-reactive protein version

**Fig. 3:**
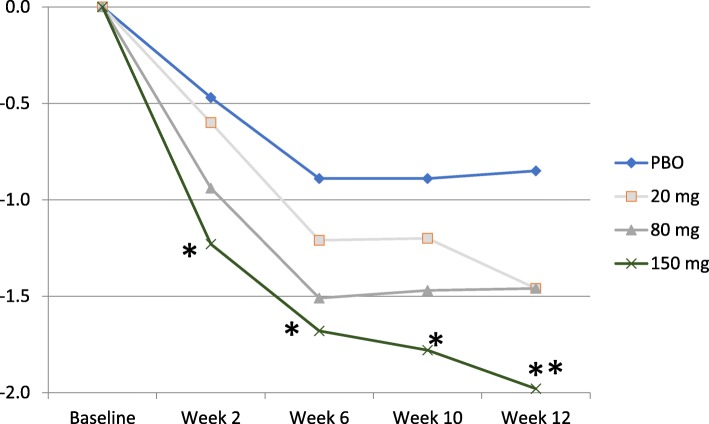
DAS28-CRP-adjusted mean change from baseline (per-protocol set). Significant *p* values compared to placebo are shown (*p* < 0.05 as one asterisk and *p* < 0.01 as two asterisks) [150 mg at week 2, *p* = 0.021; at week 6, *p* = 0.037; at week 10, *p* = 0.018; and at week 12, *p* = 0.005]. DAS28-CRP, 28-joint Disease Activity Score, C-reactive protein version; PBO placebo

DAS28-CRP scores were similar at baseline for subjects receiving placebo and those receiving namilumab (Table 2). Treatment with namilumab was associated with a clinically significant reduction in disease activity and ACR scores. At week 12, a statistically significant difference in DAS28-CRP score was seen for all doses of namilumab versus placebo both for the per-protocol analysis (*p* = 0.022) and the full analysis set (*p* = 0.015) (Fig. 2). The mean change from baseline in DAS28-CRP score at week 12 was statistically significantly different for namilumab 150 mg compared with placebo in the FAS population (*p* = 0.010). Results of the PPS analyses were consistent with the FAS analyses (*p* = 0.005 for namilumab 150 mg vs placebo). A significant separation versus placebo for this parameter was seen as early as week 2, after only 1 dose of namilumab 150 mg (*p* = 0.021) and thereafter at week 6 (*p* = 0.037) and at week 10 (*p* = 0.018). A dose-dependent response was observed over the namilumab dose range tested (Fig. 3). There was improvement in disease activity in all namilumab treatment groups, with a mean reduction from baseline to week 12 in DAS28-CRP scores versus placebo as follows: − 1.63, − 1.47, and − 1.80 for namilumab 20, 80, and 150 mg, respectively, versus − 0.99 for placebo. At week 12, the LS mean change in DAS28-CRP score from baseline was − 1.38, − 1.36, and − 1.69 for the namilumab treatment groups (20, 80, and 150 mg, respectively) versus 0.77 for placebo, which indicates improvement in disease activity, corresponding to a difference from placebo of − 0.61, − 0.59, and − 0.92 for namilumab 20, 80, and 150 mg, respectively. DAS28 changes from baseline were also analysed for MTX-IR and the TNF-IR patients, separately. The same changes were observed with MTX-IR patients (Additional file 1: Figure S1). For the TNF-IR patients, we observed a separation between placebo and 20-mg and 150-mg doses of namilumab (Additional file 2: Figure S2). However, this separation was not evident with the 80-mg dose, and this is likely due to the small sample size of the TNF-IR patients in this study, especially the sample size for the 80-mg dose cohorts (*n* = 3).

**Fig. 4:**
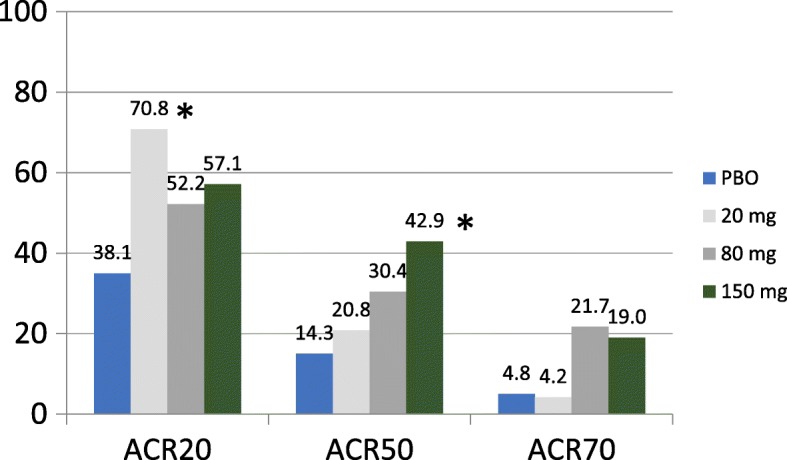
ACR20, ACR50, and ACR70 at week 12 (per-protocol set). Significant *p* values compared to placebo are shown by an asterisk (ACR20 for 20 mg, *p* = 0.031, and ACR50 for 150 mg, *p* = 0.049). ACR American College of Rheumatology, ACR20 ≥ 20% improvement, ACR50 ≥ 50% improvement, ACR70 ≥ 70% improvement, PBO placebo

**Fig. 5:**
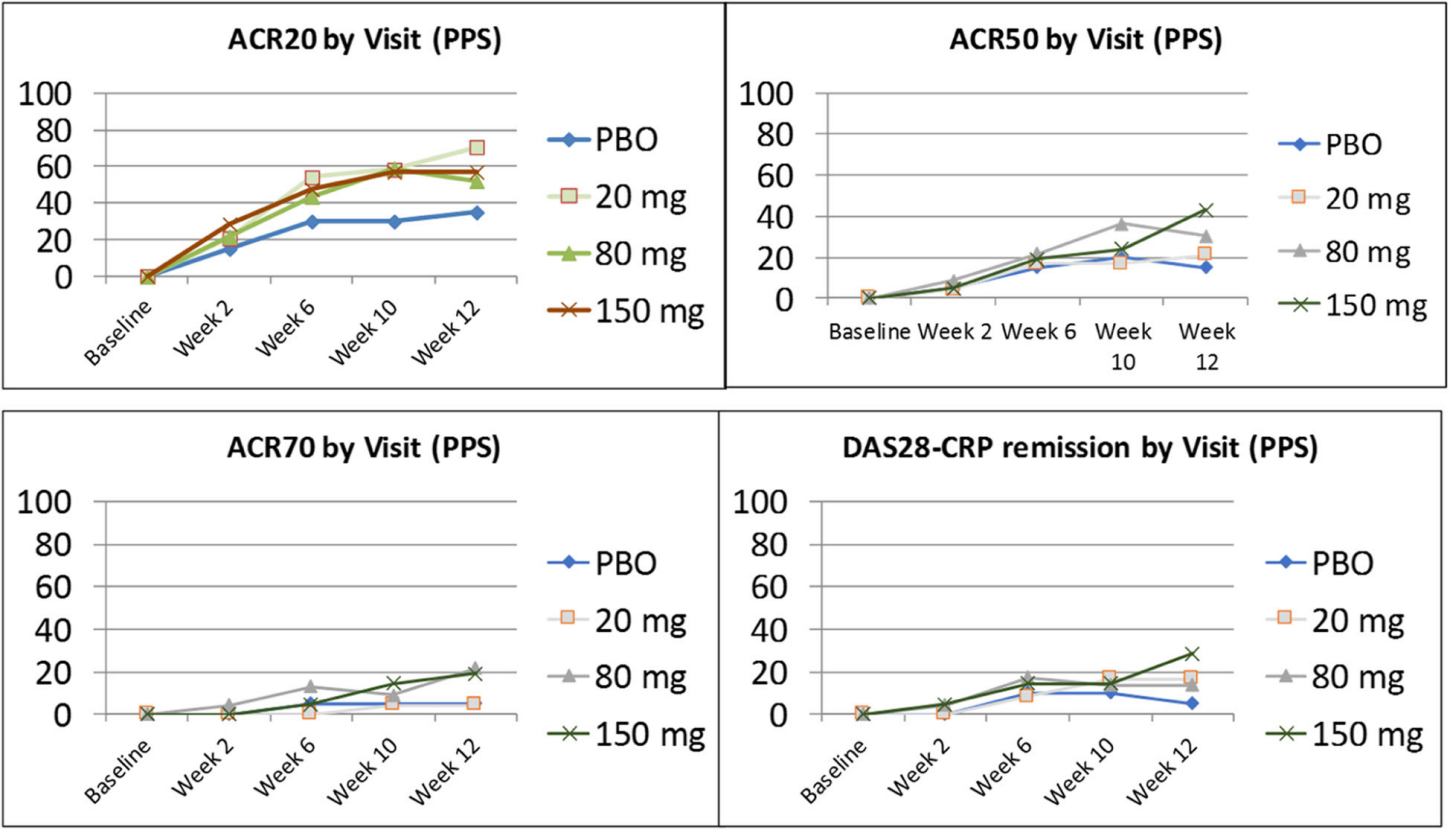
Percentage of ACR20/50/70 and DAS28-CRP remission (≤ 2.6) by study visit (PPS). ACR, American College of Rheumatology; ACR20, ≥ 20% improvement; ACR50, ≥ 50% improvement; ACR70, ≥ 70% improvement; DAS28-CRP, 28-joint Disease Activity Score, C-reactive protein version; PBO, placebo; PPS, per-protocol set. *p* value of less than 0.05 is shown by an asterisk

For the secondary endpoints of ACR categorical responses (ACR20/50/70) and DAS28-CRP remission, higher doses of namilumab showed better efficacy than placebo (Figs. 4 and 5). For the PPS, the incidence of DAS28-CRP remission (≤ 2.6) was significantly greater for the namilumab 150-mg group (26.9%) versus placebo (9%) at week 12 (*p* = 0.048). Results of the PPS were consistent with the FAS at all post-baseline visits (full analysis set (FAS) for ACR is shown in Additional file 3: Figure S3, and FAS for DAS28-CRP remission at 12 weeks is shown in Additional file 4: Figure S4).

At week 12, the proportion of subjects with ≥ 40% reduction in pain was 44.0%, 39.1%, and 30.8% for namilumab 20, 80, and 150 mg, respectively, versus 20.0% for placebo, but it did not reach statistical significance (*p* values namilumab versus placebo were 0.075, 0.151, and 0.381 for 20 mg, 80 mg, and 150 mg, respectively). At week 12, the LS mean change from baseline was − 8.55 for placebo, − 14.55 for 20 mg namilumab, − 13.6 for 80 mg, and − 13.7 for 150 mg. However, again, this did not reach statistical significance (*p* values for namilumab versus placebo were 0.055, 0.083, and 0.072 for 20 mg, 80 mg, and 150 mg, respectively). At week 12, the LS mean change from baseline in SF-36 (36-Item Short-Form Health Survey) mental health score was 7.8, 5.2, and 14.4 for namilumab 20, 80, and 150 mg, respectively, versus 3.07 for placebo. A statistically significant difference was seen between namilumab 150 mg versus placebo (*p* = 0.019). The LS mean change from baseline in SF-36 vitality score was 12.8, 12.2, and 17.3 for namilumab (20, 80, and 150 mg, respectively) versus 6.5 for placebo. A statistically significant difference was seen between namilumab 150 mg versus placebo (*p* = 0.035). There were no statistically significant differences between any of the namilumab treatment groups versus placebo for any of the other health and physical summary scores from baseline to week 12.

At week 12, EULAR good/moderate response rates for namilumab 20, 80, and 150 mg were 76.0%, 68.2%, and 76.9%, respectively, versus 45.8% for placebo. This reached statistical significance for the namilumab 20- and 150-mg treatment groups versus placebo (*p* = 0.032 and *p* = 0.025, respectively).

As previously observed [30], there were dose-dependent increases in blood GM-CSF concentrations over the namilumab dose range investigated. There was no change in GM-CSF concentrations for placebo. In addition, no clear trends were associated with namilumab treatment with regard to change in anti-citrullinated protein antibodies and rheumatoid factor versus baseline at any of the applicable visits. With regard to change from baseline in biomarkers with the potential for early detection of pulmonary alveolar proteinosis (carcinoembryonic antigen, KL-6, and SP-D), there were no trends associated with namilumab treatment. Peripheral blood cell types known to demonstrate GM-CSF responsiveness (including monocytes and macrophages, natural killer cells, neutrophils, B cells, and T cells) were assessed using flow cytometry, subtyping, and activation markers. There were no notable changes from baseline of cell types known to demonstrate GM-CSF responsiveness at any of the applicable visits.

At baseline, the majority of subjects (96.0%; 95 of 106) had negative screening results for anti-drug antibodies (ADAs). Four subjects (4.0%) had positive ADA results, with 2 (50.0%) confirmed positive and 2 (50.0%) confirmed negative in the ADA confirmatory assay. At week 12, 3 subjects (2.3%; 3 of 106) had positive ADA screening results, of which 1 (33.3%) was confirmed positive and 2 (66.7%) were confirmed negative in the ADA confirmatory assay.

**Fig. 6:**
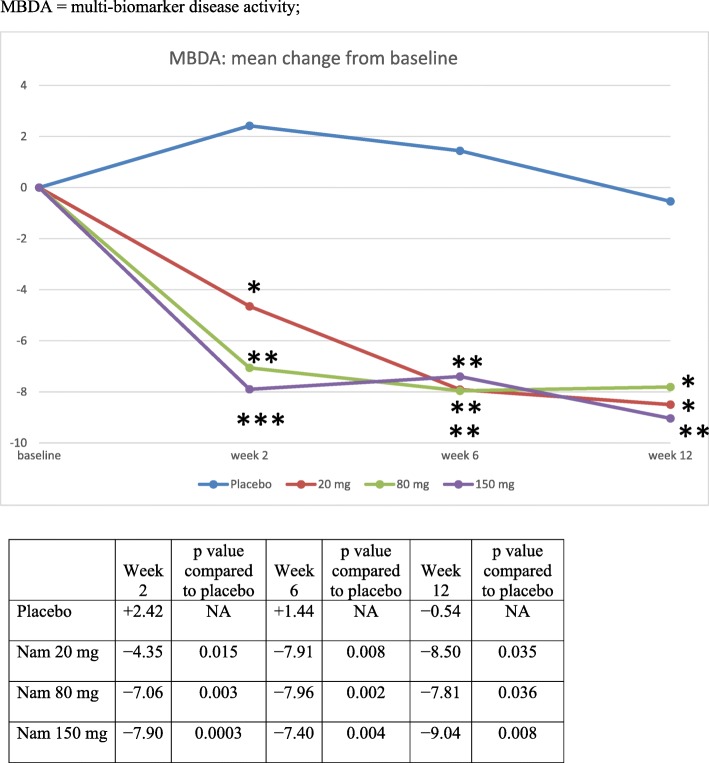
Mean change in MBDA from baseline. MBDA multibiomarker disease activity

**Fig. 7:**
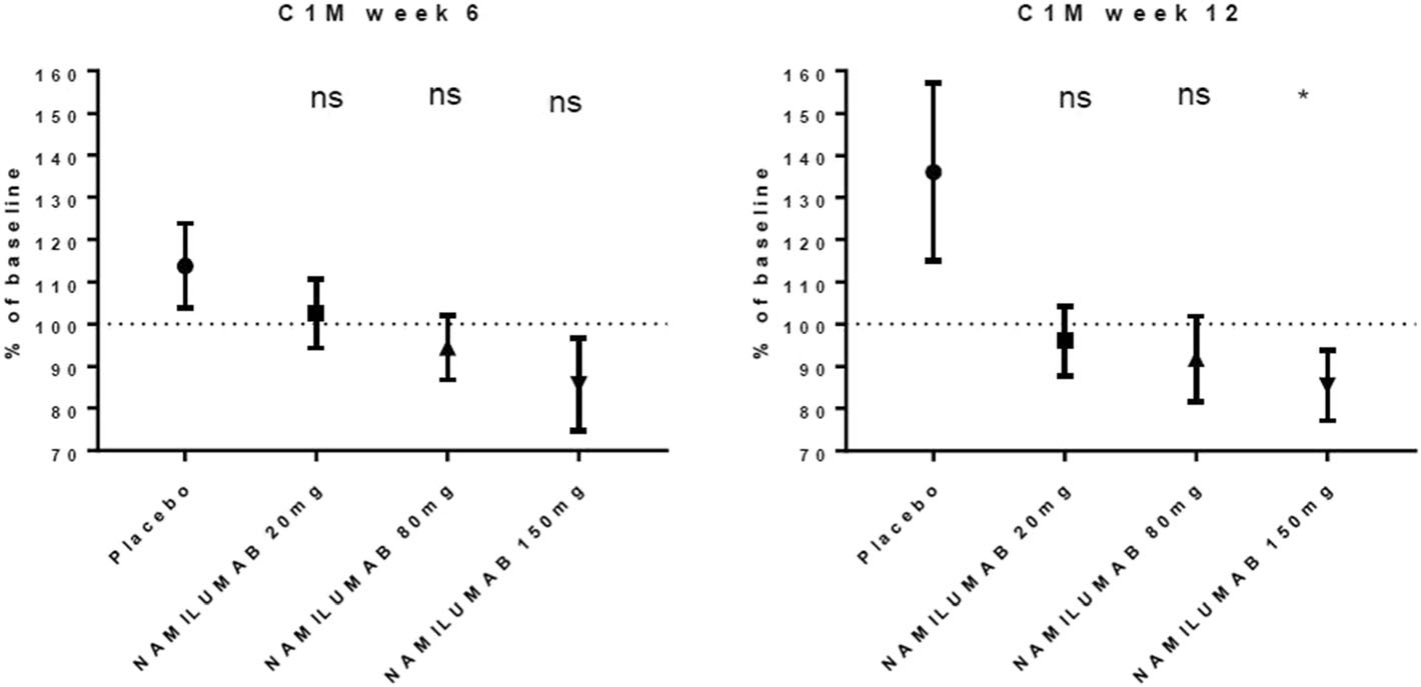
Change in C1M from baseline. Values are mean ± SE. ns not significant. *Significant *p* = 0.0227

For the total MBDA score (Fig. 6), there were highly significant reductions in all namilumab treatment arms versus placebo from week 2. At week 12, the mean decrease in MBDA score was − 0.54 for the placebo cohort and − 8.50, − 7.81, and − 9.04 for the 20-mg, 80-mg, and 150-mg cohorts, with *p* values of 0.035, 0.036, and 0.008 for the 20-mg, 80-mg, and 150-mg cohorts compared to placebo, respectively. The number of patients for whom serum samples were analysed at each time point ranged from 22 to 27, 18 to 23, 22 to 25, and 18 to 26 for namilumab 150, 80, and 20 mg and placebo, respectively. In relation to the Nordic biomarkers, there was a significant difference in change in C1M from baseline between namilumab 150 mg and placebo (*p* value 0.0227; Fig. 7). Apart from MBDA and Nordic biomarkers, there were no notable changes in biomarker parameters during the study, and no clear trends were associated with namilumab treatment.

### Safety

Over the 12-week study period, 14 of 27 subjects receiving placebo (51.9%; 25 events) and 45 of 81 receiving namilumab (55.6%; 84 events) experienced a treatment-emergent adverse event (TEAE; namilumab 20 mg, 42.8%; namilumab 80 mg, 68%; namilumab 150 mg, 57.1%). The most common TEAEs (Table 3) were nasopharyngitis (18.5%, 17.9%, 4.0%, 14.3%), dyspnoea (0.0%, 3.6%, 8.0%, 10.7%), bronchitis (7.4%, 3.6%, 4.0%, 3.6%), and headache (3.7%, 3.6%, 12.0%, 0.0%) for placebo and namilumab 20, 80, or 150 mg, respectively. Two mild AEs of urticaria developed in patients on the namilumab 20-mg arm. One AE of urticaria occurred after the first IMP injection in the abdomen: urticaria, dry mouth, and tachycardia. The patient fully recovered within 24 h without any treatment. The urticaria developed around the face and on the upper chest of the patient. The PI assessed the AE as related to the study medication and withdrew the patient from the study. The second AE of urticaria developed 2 days after the first dose and resolved 5 days later without any treatment. The study medication was maintained, and the AE did not reoccur. The PI assessed the AE as related to the study medication.

**Table 3:** Most frequent TEAEs (> 5% of subjects) in double-blind period up to 12 weeks

Preferred term	Placebo (*N* = 27)	Namilumab		
		20 mg (*N* = 28)	80 mg (*N* = 25)	150 mg (*N* = 28)
Nasopharyngitis	5 (18.5)	5 (17.9)	1 (4.0)	4 (14.3)
Dyspnoea	0	1 (3.6)	2 (8.0)	3 (10.7)
Bronchitis	2 (7.4)	1 (3.6)	1 (4.0)	1 (3.6)
Headache	1 (3.7)	1 (3.6)	3 (12.0)	0
Upper respiratory tract infection	0	0	2 (8.0)	1 (3.6)
Rheumatoid arthritis	0	2 (7.1)	2 (8.0)	0
Hypertension	0	0	0	2 (7.1)
Laryngitis	0	0	2 (8.0)	0
Menorrhagia	0	2 (7.1)	0	0
Urticaria	0	2 (7.1)	0	0

Values are *n* (%). *TEAE* treatment-emergent adverse event

One serious TEAE was reported in the namilumab 150-mg arm in a 63-year-old Japanese man who experienced a myocardial infarction on day 83 after having received the fourth scheduled and his last dose of IMP. The patient was a smoker (11 to 20 pack-years). Medical history was otherwise unremarkable. The patient was withdrawn from the study and recovered after cardiac catheterization. One additional TEAE led to study discontinuation: 1 patient initially randomized to namilumab 20 mg experienced three different AEs, on day 1, moments after the first IMP injection in the abdomen: urticaria, dry mouth, and tachycardia. The patient fully recovered within 24 h without any treatment, and therefore, this AE was qualified as severe.

Treatment-emergent adverse events of special interest which could be suggestive of pulmonary alveolar proteinosis were reported in small numbers, and their low frequency (below 5%) excluded them from Table 3. These AEs were reviewed by the pulmonary panel and DSMB and were not deemed indicative of pulmonary alveolar proteinosis but considered to be reflective an alternative diagnosis.

## Discussion

There is a growing body of evidence in support of the potential importance of the role of GM-CSF in the pathogenesis of RA. Moreover, rapid and sustained efficacy and normalization of acute-phase reactants have been consistently shown in studies targeting the GM-CSF pathway [37]. In a previous phase I study designed to assess the safety of repeated dosing in a small number of patients of subjects with RA, namilumab demonstrated preliminary evidence of efficacy [30]. This phase II trial is the first study statistically powered to evaluate the efficacy of different doses of namilumab versus placebo in a population with moderate to severe disease activity in RA. The findings show that namilumab, an investigational human monoclonal antibody targeting GM-CSF, improves the signs and symptoms of RA in such subjects. The study met its primary endpoint, and rapid and sustained improvement was demonstrated by DAS28-CRP, HAQ-DI, and ACR responses. Significant improvement was seen as early as week 2 for the highest dose (150 mg), and for all doses, scores continued to improve throughout the 12-week treatment. Of note, and a limitation of the present study, the observed placebo response rate was higher than assumed in the sample size. Despite this, the primary endpoint was met, and significant improvements were observed with namilumab over placebo. Another limitation of this phase II study was the relatively short time that subjects were exposed to the test biological. Nonetheless, although we only evaluated the effects of namilumab over 12 weeks, it was particularly encouraging that at the highest (150 mg) dose, 26.9% of subjects achieved DAS28-CRP < 2.6 (placebo 9.0%) and 42.9% showed an ACR50 response (placebo 15%). The number of subjects achieving DAS28-CRP < 2.6 or ACR50 and ACR70 responses was still rising at 12 weeks, which suggests that peak efficacy might not have been achieved. It has been shown previously that response to some biologic treatments continues to increase over the first 24 weeks of treatment, and a significant proportion of partial responders and nonresponders at week 12 can go on to achieve a clinical response with continued treatment [38]. For clinical practice, in future clinical trials, from the results of this study, 150-mg dose of namilumab should be used to offer maximum efficacy to patients, with acceptable safety. From a manufacturing point of view and tolerability for the patient as a subcutaneous injection, 150-mg dose is the maximum feasible dose at the current concentration of the antibody as the investigational product.

The safety profile was consistent with a previous phase I study of namilumab in subjects with RA [30]. Because of the link between GM-CSF and alveolar macrophage function and clearance of lung surfactant proteins, we performed intensive pulmonary lung function tests and assays for biomarkers of lung damage such as SP-D and KL-6. No meaningful differences were noted for SP-D and KL-6 between the active treatment and placebo groups. Furthermore, SP-D and KL-6 levels during the study were comparable to those described for healthy control subjects and RA patients with no interstitial lung disease [32]. No serious or opportunistic infections or severe hypersensitivity reactions were reported in this patient population during the observation period.

## Conclusion

In conclusion, the results of this study showed that namilumab induced rapid and sustained clinically significant responses in subjects with RA over the period of the study, with a favourable risk-benefit profile. The results presented in this manuscript are in line with other anti-GM-CSF antibodies and confirm the validity of the target in RA [27, 37].

## Additional files

Additional file 1: **Figure S1.** Analysis of change from baseline in DAS28-CRP score in MTX-IR patients 1a/ Graph of the mean change of DAS28 in the MTX-IR patients from baseline over the 12 weeks study duration period (DAS28-CRP = 28-joint Disease Activity Score) 1b/ table of the DAS-28 values corresponding to the graph. (DOCX 17 kb)

Additional file 2: **Figure S2.** Analysis of change from baseline in DAS28-CRP score in TNF-IR patients 1a/ Graph of the mean change of DAS28 in the TNF-IR patients from baseline over the 12 weeks study duration period (DAS28-CRP = 28-joint Disease Activity Score) 1b/ table of the DAS-28 values corresponding to the graph. (DOCX 17 kb)

Additional file 3: **Figure S3.** Data are shown for the full set analysis of outcomes for the ACR categorical responses at week 12. (DOCX 20 kb)

Additional file 4: **Figure S4.** Data are shown for the full set analysis of DAS28-CRP remission criteria at week 12. (DOCX 80 kb)

## Abbreviations

ACR: American College of Rheumatology
ADA: Anti-drug antibody
AE: Adverse event
CRP: C-reactive protein
DAS28-CRP: 28-joint Disease Activity Score, C-reactive protein version
EULAR: European League Against Rheumatism
FAS: Full analysis set
GM-CSF: Granulocyte-macrophage colony-stimulating factor
HAQ-DI: Health Assessment Questionnaire Disability Index
KL-6: Krebs von den Lungen-6
LS: Least squares
MBDA: Multibiomarker disease activity
MTX: Methotrexate therapy
MTX-IR: Inadequate response to methotrexate therapy
PPS: Per-protocol set
RA: Rheumatoid arthritis
SF-36: 36-Item Short-Form Health Survey
SP-D: Serum surfactant D
TEAE: Treatment-emergent adverse event
TNF: Tumour necrosis factor
TNF-IR: Inadequate response or intolerance to an anti-tumour necrosis factor biologic therapy
