# Supplementary material for: Efficacy and safety of namilumab, a human monoclonal antibody against granulocyte-macrophage colony-stimulating factor (GM-CSF) ligand in patients with rheumatoid arthritis (RA) with either an inadequate response to background methotrexate therapy or an inadequate response or intolerance to an anti-TNF (tumour necrosis factor) biologic therapy: a randomized, controlled trial
Source: Arthritis Res Ther. 2019 Apr 18;21:101. doi: 10.1186/s13075-019-1879-x (PMC6471864; doi:10.1186/s13075-019-1879-x)
Supplement: Supplementary file 1 — Figure S1. Analysis of change from baseline in DAS28-CRP score in MTX-IR patients 1a/ Graph of the mean change of DAS28 in the MTX-IR patients from baseline over the 12 weeks study duration period (DAS28-CRP = 28-joint Disease Activity Score) 1b/ table of the DAS-28 values corresponding to the graph. (DOCX 17 kb) [file 13075_2019_1879_MOESM1_ESM.docx]

**Figure S1: Analysis of Change from Baseline in DAS28-CRP Score in MTX-IR patients**

|  | Placebo | Nam 20 mg | Nam 80 mg | Nam 150 mg |
| --- | --- | --- | --- | --- |
| n | 23 | 23 | 22 | 24 |
| baseline | 0 | 0 | 0 | 0 |
| week 2 | -0.49 | -0.62 | -0.81 | -0.99 |
| week 6 | -0.93 | -1.4 | -1.49 | -1.46 |
| week 10 | -1.05 | -1.47 | -1.64 | -1.56 |
| week 12 | -1.1 | -1.59 | -1.62 | -1.49 |
