# Supplementary material for: Efficacy and safety of namilumab, a human monoclonal antibody against granulocyte-macrophage colony-stimulating factor (GM-CSF) ligand in patients with rheumatoid arthritis (RA) with either an inadequate response to background methotrexate therapy or an inadequate response or intolerance to an anti-TNF (tumour necrosis factor) biologic therapy: a randomized, controlled trial
Source: Arthritis Res Ther. 2019 Apr 18;21:101. doi: 10.1186/s13075-019-1879-x (PMC6471864; doi:10.1186/s13075-019-1879-x)
Supplement: Supplementary file 2 — Figure S2. Analysis of change from baseline in DAS28-CRP score in TNF-IR patients 1a/ Graph of the mean change of DAS28 in the TNF-IR patients from baseline over the 12 weeks study duration period (DAS28-CRP = 28-joint Disease Activity Score) 1b/ table of the DAS-28 values corresponding to the graph. (DOCX 17 kb) [file 13075_2019_1879_MOESM2_ESM.docx]

**Figure S2**

DAS28 data presented for TNF-IR patients

2a/ DAS28 Graph for TNF-IR

2b/ DAS28 values over the 12 weeks

|  | Placebo | Nam 20 mg | Nam 80 mg | Nam 150 mg |
| --- | --- | --- | --- | --- |
| n | 4 | 5 | 3 | 4 |
| baseline | 0 | 0 | 0 | 0 |
| week 2 | 0 | -0.73 | -0.7 | -0.36 |
| week 6 | -0.4 | -0.83 | -0.9 | -1.56 |
| week 10 | -0.32 | -1.2 | -0.8 | -1.81 |
| week 12 | -0.31 | -1.97 | -0.58 | -1.93 |
