# Supplementary material for: Efficacy and safety of namilumab, a human monoclonal antibody against granulocyte-macrophage colony-stimulating factor (GM-CSF) ligand in patients with rheumatoid arthritis (RA) with either an inadequate response to background methotrexate therapy or an inadequate response or intolerance to an anti-TNF (tumour necrosis factor) biologic therapy: a randomized, controlled trial
Source: Arthritis Res Ther. 2019 Apr 18;21:101. doi: 10.1186/s13075-019-1879-x (PMC6471864; doi:10.1186/s13075-019-1879-x)
Supplement: Supplementary file 3 — Figure S3. Data are shown for the full set analysis of outcomes for the ACR categorical responses at week 12. (DOCX 20 kb) [file 13075_2019_1879_MOESM3_ESM.docx]

**Figure S3: Full set analysis of ACR clinical response at week 12.**

|  | Placebo | Nam 20 mg | Nam 80 mg | Nam 150 mg |
| --- | --- | --- | --- | --- |
| ACR 20 | 40 | 72 | 52.2 | 53.8 |
| P-value |  | 0.025 | 0.399 | 0.324 |
| ACR 50 | 16 | 20 | 30.4 | 38.5 |
| P-value |  | 0.713 | 0.241 | 0.08 |
| ACR 70 | 8 | 4 | 21.7 | 15.4 |
| P-value |  | 0.559 | 0.194 | 0.421 |

*
