# Supplementary material for: Efficacy and safety of namilumab, a human monoclonal antibody against granulocyte-macrophage colony-stimulating factor (GM-CSF) ligand in patients with rheumatoid arthritis (RA) with either an inadequate response to background methotrexate therapy or an inadequate response or intolerance to an anti-TNF (tumour necrosis factor) biologic therapy: a randomized, controlled trial
Source: Arthritis Res Ther. 2019 Apr 18;21:101. doi: 10.1186/s13075-019-1879-x (PMC6471864; doi:10.1186/s13075-019-1879-x)
Supplement: Supplementary file 4 — Figure S4. Data are shown for the full set analysis of DAS28-CRP remission criteria at week 12. (DOCX 80 kb) [file 13075_2019_1879_MOESM4_ESM.docx]

**Figure S4: Full set analysis of DAS28-CRP remission at week 12.**

|  | Placebo | Nam 20 mg | Nam 80 mg | Nam 150 mg |
| --- | --- | --- | --- | --- |
| DAS28 CRP Remission | 8 | 16 | 13 | 26.9 |
|  | P-value | 0.393 | 0.571 | 0.093 |
